# Synthesis, anticholinesterase and antioxidant potentials of ketoesters derivatives of succinimides: a possible role in the management of Alzheimer’s

**DOI:** 10.1186/s13065-015-0107-2

**Published:** 2015-05-26

**Authors:** Abdul Sadiq, Fawad Mahmood, Farhat Ullah, Muhammad Ayaz, Sajjad Ahmad, Faizan Ul Haq, Ghazan Khan, Muhammad Saeed Jan

**Affiliations:** Department of Pharmacy, University of Malakand, Chakdara, Dir Pakistan; Department of Pharmacy, Sarhad University of Science and Information Technology, Peshawar, KPK Pakistan

**Keywords:** Michael addition, Ketoesters, Succinimides, Acetylcholinesterase, Antioxidant, Alzheimer’s

## Abstract

**Background:**

Based on the pharmacological potency and structural features of succinimides, this study was designed to synthesize new ketoesters derivatives of succinimides. Furthermore, the synthesized compounds were evaluated for their possible anticholinesterase and antioxidant potentials. The compounds were synthesized by organocatalytic Michael additions of α-ketoesters to *N*-aryl maleimides. Acetyl and butyrylcholinesterase inhibitory activities were determined using Ellman’s spectrophotometric assay. The antioxidant activity was performed with DPPH and ABTS free radicals scavenging assay.

**Results:**

The Michael additions of α-ketoesters to maleimides was promoted by 8-hydroxyquinoline. The organocatalyst (8-hydroxyquinoline, 20 mol %) produced the compounds in relatively shorter time (20–24 h) and with excellent isolated yields (84-98 %). The synthesized compounds (1–4) showed outstanding acetylcholinesterase (AChE) and butyrylcholinesterase (BChE) inhibitory potentials, *i.e.*, 98.75 and 90.00 % respectively for compound 2, with IC_50_ < 0.1 μg/mL. Additionally, compounds 1–4 revealed moderate antioxidant activity at different concentrations. In DPPH free radical scavenging assay, compound 1 showed dominant result with 72.41 ± 0.45, 52.49 ± 0.78 and 35.60 ± 0.75 % inhibition at concentrations of 1000, 500 and 250 μg/mL respectively, IC_50_ value of 440 μg/mL. However, the free radical scavenging was better when used ABTS free radicals. In ABTS free radicals scavenging assay compound 1 exhibited 88.51 ± 0.62 % inhibition at highest tested concentration *i.e.*, 1000 μg/mL.

**Conclusions:**

Herein, we have synthesized four ketoesters derivatives of succinimides in a single step reaction and high yields. As a highlight, we have showed a first report on the anticholinesterase and antioxidant potentials of succinimides. All the compounds showed overwhelming enzyme inhibitions and moderate antioxidant potentials.

**Graphical Abstract:**
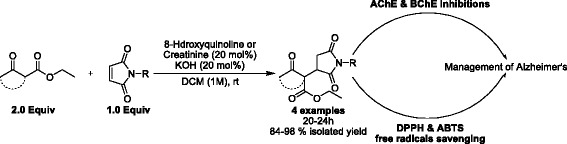
Graphical representation of synthesis, anticholinesterase and antioxidant potentials of ketoester derivatives of succinimides.

**Electronic supplementary material:**

The online version of this article (doi:10.1186/s13065-015-0107-2) contains supplementary material, which is available to authorized users.

## Background

**Fig. 1:**
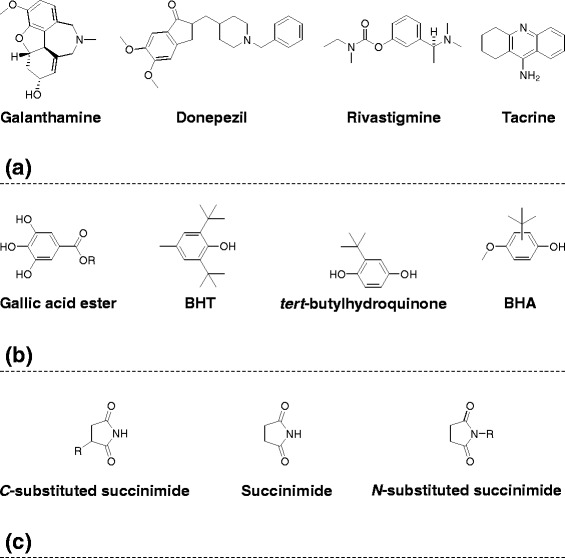
**a** Natural and synthetic cholinesterase inhibitors; (**b**) Commonly employed synthetic antioxidants; (**c**) General structures of succinimide and its derivatives

Alzheimer’s disease (AD), a chronic neurodegenerative disorder is the most common dementia effecting a greater number of elder population worldwide [1]. Several biochemical pathways are known for the management of AD but the most important one is to inhibit a vital neurotransmitter responsible for signal transfer and cognitive functions [2]. Inhibition of acetylcholine (ACh), the neurotransmitter, can restore the level of ACh in the synaptic region and thus reinstate deficient cholinergic neurotransmission [3]. In synaptic region, the acetylcholine hydrolyzes giving choline and acetyl group with the help of biocatalyst acetylcholinesterase (AChE) and butyrylcholinesterase (BChE) [4]. The inhibitions of AChE and BChE is the key target in the management of AD [5]. Several natural and synthetic cholinesterase inhibitors like galanthamine, donepezil, rivastigmine and tacrine (Fig. 1a) are reported but their use is limited due to severe side effects and low efficiency [6]. Therefore, a main goal of the current researchers is the development of novel, safe, effective and economical drug candidates in the management of neurological disorders.

In the last decade, it has been reported that AD is associated with inflammatory process in which reactive oxygen species (ROS) are produced in the body [7]. The ROS are able to damage biomolecules like enzymes, lipids, proteins, DNA and RNA leading to inflammation [8]. To cope with the situation, human body has the ability to follow several defense mechanisms including enzymatic and non-enzymatic antioxidant pathways [9]. However, this is practically impossible for the human body to scavenge all ROS and attenuate inflammation processes [10]. Eventually, the excess of ROS leads to the progression of chronic diseases like cardiovascular diseases, cancer, diabetes, nephritis, rheumatism and aging [11]. Therefore natural and synthetic antioxidants are used employed in the management of neurological disorders like AD [12]. The natural antioxidants are being common in herbs, fruits and vegetables [13, 14]. Synthetic antioxidants (Fig. 1b) are gallic acid esters, butylated hydroxy toluene (BHT), tertiary butylated hydroquinone and butylated hydroxy anisole (BHA) [15]. Both natural and synthetic antioxidants are associated with certain limitations like geographical distribution [16] and seasonal availability [17] of the natural sources and adverse side effects of synthetic antioxidants [18]. Therefore, a constant research is needed in the field to find out safe and cost effective antioxidants.

Succinimides, a known class of anticonvulsant drugs have been reported to posses several other pharmacological potentials [19]. The basic nuclei of succinimide is “pyrrolidine-2,5-dione”, *i.e.* a five member ring with a nitrogen atom and two carbonyl groups as shown in Fig. 1c. However, the basic skeleton can be modified to C or N-substituted succinimides with different alkyl or aryl groups which can lead to potential drug molecules [20–23]. The commonly employed natural and synthetic drugs in the treatment of AD possess nitrogen atom, aromatic ring and/or carbonyl group in common in their structure. Similarly, the compounds used in the scavenging of free radicals must possess electron rich groups like hydroxyl or conjugation in their structure as shown in Fig. 1 (a & b).

## Results and discussion

### Synthesis of compounds (1–4)

Herein, we have synthesized four new ketoester derivatives of succinimides (1–4) by organocatalytic Michael additions of α-ketoesters to *N*-phenyl and *N*-benzyl maleimides. A number of available nitrogenous compounds were screened as achiral organocatalysts for the catalysis of these reactions. Among them creatinine and 8-hydroxyquinoline promote the reactions in relatively shorter time with even low mol % loading. All the compounds were synthesized in a reasonable reaction time (less than 24 h) and with high isolated yields (84–98 %) as shown in Table 1.

**Table 1:** Synthesized ketoester derivatives of succinimides

Product No	Structure	Time^[a]^ (h)	Yield^[b]^ (%)
1		20	95
2		24	91
3		22	98
4		23	84

[a] Time of reaction completion; [b] Isolated yield after column chromatography

### Acetylcholinesterase inhibition assay

The purified products were evaluated for anticholinesterase potentials (AChE & BChE) using Ellman’s assay as shown in Tables 2 and 3 respectively.

**Table 2:** Acetylcholinesterase inhibition of compounds 1-4

Compounds	Concentration	Percent AChEI	IC_50_
	(μg/mL)	(mean ± SEM)	(μg/mL)
1	1000	98.00 ± 0.70^ns^	<0.1
	500	94.25 ± 0.05 ^ns^	
	250	93.25 ± 0.25 ^ns^	
2	1000	98.75 ± 0.25 ^ns^	<0.1
	500	91.75 ± 0.04 ^ns^	
	250	91.00 ± 0.52 ^ns^	
3	1000	98.50 ± 0.09 ^ns^	<0.1
	500	96.00 ± 0.80 ^ns^	
	250	95.25 ± 0.20 ^ns^	
4	1000	97.25 ± 0.07 ^ns^	<0.1
	500	96.00 ± 0.55 ^ns^	
	250	93.25 ± 0.15 ^ns^	
Galanthamin	1000	94.22 ± 1.01	<0.1
e	500	92.28 ± 0.43	
	250	85.35 ± 0.83	

Data is represented as mean ± SEM, *n* = 3

Two-way ANOVA followed by Bonferroni test was applied for significant difference between standard drugs and test samples at 95 % confidence interval. Values significantly not different in comparison to standard drug

**Table 3:** Butyrylcholinesterase inhibition of compounds 1-4

Compounds	Concentration	Percent AChEI	IC_50_
	(μg/mL)	(mean ± SEM)	(μg/mL)
1	1000	90.25 ± 0.02 ^ns^	42
	500	82.50 ± 0.18 ^ns^	
	250	72.50 ± 0.02 ^ns^	
2	1000	90.00 ± 0.10 ^ns^	<0.1
	500	86.75 ± 0.22 ^ns^	
	250	84.25 ± 0.12 ^ns^	
3	1000	89.25 ± 0.50 ^ns^	7
	500	89.00 ± 0.30 ^ns^	
	250	78.25 ± 0.04 ^ns^	
4	1000	98.50 ± 0.18 ^ns^	2
	500	88.50 ± 0.50 ^ns^	
	250	87.50 ± 0.04 ^ns^	
Galanthamin	1000	94.50 ± 0.71	53
e	500	85.47 ± 0.59	
	250	71.72 ± 0.51	

Data is represented as mean ± SEM, *n* = 3

Two-way ANOVA followed by Bonferroni test was applied for significant difference between standard drugs and test samples at 95 % confidence interval. Values significantly not different in comparison to standard drug

The compounds (1–4) exhibited outstanding acetylcholinesterase inhibitions (AChEI) potentials at three tested concentrations (1000, 500 and 250 μg/mL) as shown in Table 2. The highest observed acetylcholinesterase inhibition was with compound 2 at concentration of 1000 μg/mL exhibiting 98.75 ± 0.25 % activity, IC_50_ < 0.1 μg/mL. Decreasing the concentration of **2** to 500 μg/mL and then to 250μg/mL dropped the percent inhibitions to 91.75 ± 0.04 and 91.00 ± 0.52 % respectively. Similarly, at the highest tested concentration (1000 μg/mL) compounds 1, 3 and 4 revealed 98.00 ± 0.70, 98.50 ± 0.09 and 97.25 ± 0.07 % inhibitions respectively with IC_50_ of <0.1 μg/mL. Our compounds were comparatively potent to the standard drug galanthamine which reveal 94.22 ± 0.01, 92.28 ± 0.43 and 85.35 ± 0.83 % AChE inhibition at 1000, 500 and 250 μg/mL concentrations respectively with IC_50_ < 0.1 μg/mL.

### Butyrylcholinesterase inhibition assay

The butyrylcholinesterase inhibitions (BChEI) of compounds 1–4 are summarized in Table 3. In BChEI, compound 2 proven to be most potent with IC_50_ value of <0.1 μg/mL inhibiting 90.00 ± 0.10, 86.75 ± 0.22 and 84.25 ± 0.12 % BChE at concentrations of 1000, 500 and 250 μg/mL respectively. The percent BChEI potentials of the remaining three compounds were in an order of 4 > 3 > 1 with IC_50_ values of 2, 7 and 42 μg/mL respectively as shown in Table 3. In comparison to galanthamine (positive control) all of our four compounds (1–4) reached to a similar level of AChE and BChE inhibitions.

### DPPH free radicals scavenging assay

Antioxidant activity for the synthesized compounds were evaluated using 1,1-diphenyl 2-picrylhydrazyl (DPPH) and 2,2-azinobis[3-ethylbenzthiazoine]-6-sulfonic acid (ABTS) as free radical sources. In DPPH free radicals scavenging assay (Table 4), compound 1 showed a better activity (72.41 ± 0.45 %) followed by 2, 3 and 4 with 70.32 ± 0.61, 60.40 ± 0.49 and 45.80 ± 0.61 % free radicals scavenging respectively at highest tested concentration. At the same tested concentration (1000 μg/mL), positive control ascorbic acid reached to 93.56 ± 0.37 % free radicals scavenging with IC_50_ 20 μg/mL.

**Table 4:** DPPH free radicals scavenging assay of compounds 1-4

Compounds	Conc. (μg/mL)	Percent inhibition (mean ± SEM)	IC_50_ (μg/mL)
1	1000	72.41 ± 0.45***	440
	500	52.49 ± 0.78***	
	250	35.60 ± 0.75***	
2	1000	70.32 ± 0.61***	460
	500	52.48 ± 0.56***	
	250	33.61 ± 0.66***	
3	1000	60.40 ± 0.49***	535
	500	54.64 ± 0.70***	
	250	30.59 ± 0.67***	
4	1000	45.80 ± 0.61***	>1000
	500	24.75 ± 0.64***	
	250	20.34 ± 0.58***	
Ascorbic	1000	93.56 ± 0.37	20
acid	500	81.71 ± 0.54	
	250	78.61 ± 0.23	

Data is represented as mean ± SEM, *n* = 3

Two-way ANOVA followed by Bonferroni test was applied for significant difference between standard drugs and test samples at 95 % confidence interval. Values significantly different as compare to positive control, ***P <0.001

### ABTS free radicals scavenging assay

The free radicals scavenging activity was slightly better when using ABTS free radicals (Table 5). The potency of compounds in ABTS free radicals scavenging activity was in an order of 3 > 1 > 2 > 4 with IC_50_ values of 73, 90, 141 and 173 μg/mL respectively. Ascorbic acid scavenge 91.62 ± 0.62, 87.23 ± 0.47 and 84.66 ± 0.88 % ABTS free radicals at concentrations of 1000, 500 and 250 μg/mL respectively with IC_50_ < 0.1 μg/mL.

**Table 5:** ABTS free radicals scavenging assay of compounds 1-4

Compounds	Conc. (μg/mL)	Percent inhibition (mean ± SEM)	IC_50_ (μg/mL)
1	1000	88.51 ± 0.62**	90
	500	77.45 ± 0.54***	
	250	65.00 ± 0.57***	
2	1000	86.49 ± 0.49***	141
	500	72.45 ± 0.65***	
	250	59.66 ± 0.66***	
3	1000	80.41 ± 0.73***	73
	500	71.25 ± 0.48***	
	250	64.67 ± 0.89***	
4	1000	73.59 ± 0.43***	173
	500	67.33 ± 0.77***	
	250	54.00 ± 1.15***	
Ascorbic	1000	91.62 ± 0.62	<0.1
acid	500	87.23 ± 0.47	
	250	84.66 ± 0.88	

Data is represented as mean ± SEM, *n* = 3

Two-way ANOVA followed by Bonferroni test was applied for significant difference between standard drugs and test samples at 95 % confidence interval. Values significantly different as compare to positive control, **P < 0.01 and ***P < 0.001

Organocatalysis is an emerging field in practice over the past decade [24]. Over this time different organocatalysts have been explored for various organic reactions [25]. Michael addition is one of the important reactions targeted by several researchers for testing potent organocatalysts [26]. In Michael addition reactions, maleimide is an emerging acceptor substrate. The first report on Michael addition of ketoesters to maleimides is published in 2006 and since then such type of products are uncommon in literature [27].

Medicinally, succinimides are vital drug candidates and building blocks for natural products like δ-lactams [28]. Various methods are available for the synthesis of succinimides, but due to the emerging trend of organocatalytic reactions, Michael additions is a currently employing method [29]. Asymmetric Michael additions of aldehydes [20], ketones [21], cyanoacetates [22] and ketoesters [23] derivatives have been reported. However, most of the synthesized succinimides are unexplored biologically and pharmacologically. To the best of our literature search, ketoesters derivatives of succinimides are not reported for anticholinesterase and antioxidant potentials. Based on the gapes in the published literature and the structural features of the available anticholinesterase and antioxidant drugs (Fig. 1) this study was designed to evaluate the ketoester derivatives of succinimides for anticholinesterase and antioxidant potentials.

As obvious from Fig. 1a that the commonly employed cholinesterase inhibitors possess nitrogen atom, aromatic ring and/or carbonyl group in their structure. In determining the acetyl and butyrylcholinesterase inhibitions potentials our compounds reached to an excel level of activity. A possible reason for this overwhelming cholinesterase inhibition might be the structural features similarities with the commonly employed drugs as shown in Fig. 1 (a & c). However, the commonly employed antioxidants possess electron rich groups like hydroxyl and an aromatic ring, as shown in Fig. 1b. All of our compounds (1–4) contain aromatic ring in their main structures but lack the hydroxyl groups. This structural conflict ultimately resulted in moderate to poor free radicals scavenging. Moreover, all of our four compounds have almost similar structural units with very minor changes therefore they exhibited almost a similar level of individual activities like anticholinesterase and antioxidant potentials.

## Experimental

### General information and instrumentation

All the chemical reactions were set up in 2.0 mL reaction vial with cap. Liquid reagents were transferred with syringes. TLC analysis was performed for routine monitoring of all the reactions. The TLC plates were precoated of silica gel 60 F_254_ and visualized under UV lamp or iodine stain. All column chromatography were performed with analytical grade silica gel (0.040-0.063 mm). *n*-Hexane and ethyl acetate were used for column chromatography.

NMR spectra were recorded on JEOL ECX 400 spectrometer, operating at 400 MHz for ^1^H and 100 MHz for ^13^C. Chemical shifts (δ) were reported in parts per million (ppm) downfield from tetramethylsilane (TMS = 0). Multiplicities are abbreviated as: (s = singlet, d = doublet, t = triplet, q = quartet, br = broad, m = multiplet). Coupling constants are expressed in Hz. FT-IR spectra were obtained on Nicolet Avatar 370 thermonicolet spectrometer. MS data was measured on a Bruker Daltonics HCT Ultra. HRMS were recorded on a Brukar micrOTOF instrument with an ionization potential of 70 eV with ESI positive mode.

#### Ethyl 2-oxo-1-(2,5-dioxo-1-phenylpyrrolidin-3-yl)cyclopentanecarboxylate (1)

Ethyl 2-oxocyclopentanecarboylate (2 mmol, 296.40 μl) in combination with 8-hydroxyquinoline (20 mol %, 29.03 mg) and potassium hydroxide (20 mol %, 11.20 mg) was added to a small vial containing 1.0 mL of DCM. After stirring for 2 min added *N*-phenylmaleimide (1 mmol, 173 mg). The reaction was stirred at room temperature and the progress of reaction was monitored by TLC. The reaction got completed in 20 h. The reaction was stopped and diluted with H_2_O (15 mL) and extracted with dichloromethane (3 × 15 mL). All the organic layers were combined and dried with anhydrous sodium sulfate (Na_2_SO_4_), filtered, and concentrated at reduced pressure using rotary evaporator. The crude reaction mixture was purified by column chromatography. The isolated yield of the pure product was calculated after complete drying which was 95 % (312 mg).

^1^H NMR (400 MHz, CDCl_3_) (ppm): 1.16-1.20 (m, 3H), 1.71-2.22 (m, 2H), 2.35-2.40 (m, 3H), 2.40-2.70 (m, 2H), 2.94 (dd, *J* = 9.5, 18.3 Hz, 1H), 3.02-3.07 (m, 1H), 4.02-4.20 (m, 2H), 7.14-7.22 (m, 2H), 7.26-7.38 (m, 3H); (^1^H NMR provided in additional file 1) ^13^C NMR (100 MHz, CDCl_3_) (ppm): 12.97, 13.14, 18.65, 19.91, 26.37, 31.56, 31.61, 36.95, 37.01, 53.76, 59.96, 60.28, 61.15, 125.54, 127.60, 128.10, 130.77, 130.96, 168.44, 169.17, 173.57, 174.00, 175.49, 211.47, 211.89, 213.00; (^13^C NMR provided in Additional file 1) FT-IR (KBr) v_max_: 3435, 3021, 1745, 1720, 1658, 1646, 1283, 1108, 1035 cm^1^; MS (EI), m/z (relative intensity): 352 [M + Na]^+^ (100 %); HRMS (ESI-TOF): Calculated for C_18_H_19_NO_5_ [M + Na]^+^ 352.1161; found: 352.1149; R_f_ = 0.44 (EtOAc/n-Hex 1:5).

#### Ethyl 1-(1-benzyl-2,5-dioxopyrrolidin-3-yl)-2-oxocyclopentanecarboxylate (2) [27]

Added ethyl 2-oxocyclopentanecarboxylate (2 mmol, 295.80 μl) to dichloromethane (1.0 mL) followed by 8-hydroxyquinoline (20 mol %, 29.03 mg) and potassium hydroxide (20 mol %, 11.20 mg) and stir for 2 min. Then added *N*-benzylmaleimide (1 mmol, 187.19 mg) to the reaction and continue stirring at room temperature. The progress of the reaction was monitored by TLC. Full conversion of the starting material was observed in 24 h. The reaction was stopped and diluted with H_2_O (15 mL) and extracted with dichloromethane (3 × 15 mL). All the organic layers were combined and dried with anhydrous sodium sulfate (Na_2_SO_4_), filtered, and concentrated at reduced pressure using rotary evaporator. The crude reaction mixture was purified by column chromatography. The isolated yield of the pure product was calculated after complete drying which was 91 % (312 mg).

^1^H NMR (400 MHz, CDCl_3_) (ppm): 1.20 (t, *J* = 7.1 Hz, 3H), 1.73-1.84 (m, 1H), 1.93-2.14 (m, 2H), 2.19-2.25 (m, 2H), 2.30-2.63 (m, 2H), 2.76-2.87 (m, 1H), 3.07 (t, *J* = 9.0 Hz, 1H), 4.02-4.14 (m, 4H), 7.09-7.23 (m, 3H), 7.28-7.40 (m, 2H); (^1^H NMR provided in Additional file 1) ^13^C NMR (100 MHz, CDCl_3_) (ppm): 9.33, 9.40, 14.66, 18.13, 18.18, 21.43, 26.91, 27.02, 37.67, 45.93, 53.58, 56.78, 58.16, 62.40, 60.08, 116.60, 120.42, 120.66, 128.14, 128.63, 137.12, 177.02, 184.58, 186.81, 217.81; R_f_ = 0.53 (EtOAc/n-Hex 1:5). (^13^C NMR provided in Additional file 1).

#### Ethyl 2-oxo-1-(2,5-dioxo-1-phenylpyrrolidin-3-yl)cyclohexanecarboxylate (3)

To a stirred reaction containing ethyl-2-oxocyclohexanecarboxylate (2 mmol, 319.90 μl), creatinine (20 mol %, 22.60 mg) and potassium hydroxide (20 mol %, 11.20 mg) in DCM (1.0 mL) was added *N*-phenylmaleimide (1 mmol, 173.17 mg). The reaction was continued at room temperature and progress was monitored by using TLC. The reaction got completed in 22 h. The reaction was stopped and diluted with H_2_O (15 mL) and extracted with dichloromethane (3 × 15 mL). All the organic layers were combined and dried with anhydrous sodium sulfate (Na_2_SO_4_), filtered, and concentrated at reduced pressure using rotary evaporator. The crude reaction mixture was purified by column chromatography. The isolated yield of the pure product was calculated after complete drying which was 98 % (336 mg).

^1^H NMR (400 MHz, CDCl_3_) (ppm): 0.85 (t, *J* = 7.5 Hz, 3H), 1.18-1.39 (m, 6H), 1.58-1.70 (m, 2H), 2.40-2.47 (m, 1H), 2.60-2.82 (m, 2H), 4.11-4.19 (m, 2H), 7.19-7.42 (m, 3H), 7.46 (dd, *J* = 3.4, 5.7, 1H), 7.64 (dd, *J* = 3.3, 5.8, 1H); (^1^H NMR provided in Additional file 1) ^13^C NMR (100 MHz, CDCl_3_) (ppm): 12.93, 13.01, 13.21, 17.23, 18.87, 23.37, 36.88, 39.99, 41.13, 52.95, 59.24, 59.79, 60.14, 60.45, 61.45, 126.15, 126.24, 127.08, 128.64, 130.04, 167.98, 169.26, 173.21, 174.82, 212.05, 213.70; (^13^C NMR provided in additional file 1) FT-IR (KBr) v_max_: 3422, 2947, 1754, 1655, 1628, 1608, 1442, 1403, 1246, 1137, 927 cm^1^; MS (EI), m/z (relative intensity): 366 [M + Na]^+^ (100 %); HRMS (ESI-TOF): Calculated for C_19_H_21_NO_5_ [M + Na]^+^ 366.1317; found: 366.1312; R_f_ = 0.55 (EtOAc/n-Hex 1:5).

#### 1Ethyl 1-(1-benzyl-2,5-dioxopyrrolidin-3-yl)-2-oxocyclohexanecarboxylate (4)

The reaction was started by addition of ethyl 2-oxocyclohexanecarboxylate (2 mmol, 319.90 μl) to DCM (1.0 mL) containing creatinine (20 mol %, 22.60 mg) and potassium hydroxide (20 mol %, 11.20 mg) in a vial. After 2 min of stirring, *N*-benzylmaleimide (187.19 mg) was continued the reaction at room temperature. TLC was used to monitor the progress of reaction. The reaction completed in 23 h. The reaction was stopped and diluted with H_2_O (15 mL) and extracted with dichloromethane (3 × 15 mL). All the organic layers were combined and dried with anhydrous sodium sulfate (Na_2_SO_4_), filtered, and concentrated at reduced pressure using rotary evaporator. The crude reaction mixture was purified by column chromatography. The isolated yield of the pure product was calculated after complete drying which was 84 % (300 mg).

^1^H NMR (400 MHz, CDCl_3_) (ppm): 0.85 (t, *J* = 7.5 Hz, 3H), 1.18-1.37 (m, 4H), 1.57-1.68 (m, 2H), 1.72-1.78 (m, 1H), 2.40-2.47 (m, 1H), 2.57 (dd, *J* = 9.2, 18.0 Hz, 1H), 2.65-2.73 (m, 1H), 3.12 (dd, *J* = 6.0, 9.2 Hz, 1H), 4.10-4.20 (m, 2H), 4.52-4.67 (m, 2H), 7.18-7.28 (m, 3H), 7.30-7.33 (m, 2H); (^1^H NMR provided in additional file 1) ^13^C NMR (100 MHz, CDCl_3_) (ppm): 9.93, 13.04, 13.11, 20.59, 21.95, 25.59, 2933, 31.35, 31.90, 37.70, 39.71, 41.38, 42.76, 61.17, 61.61, 62.07, 67.13, 126.70, 127.59, 127.62, 127.78, 129.86, 131.43, 134.73, 166.73, 174.23, 175.82, 176.34, 205.59, 205.75; (^13^C NMR provided in Additional file 1) FT-IR (KBr) v_max_: 3430, 2911, 1760, 1700, 1625, 1590, 1480, 1428, 1370, 1230, 1180, 1025, 915, 680 cm^1^; MS (EI), m/z (relative intensity): 380 [M + Na]^+^ (100 %); HRMS (ESI-TOF): Calculated for C_20_H_23_NO_5_ [M + Na]^+^ 380.1474; found: 380.1500; R_f_ = 0.63 (EtOAc/n-Hex 1:5).

## Conclusions

In conclusion, we synthesize new ketoesters derivatives of succinimides. All the compounds were synthesized in a single step reaction and with high isolated yields. The highlight of the work was the first report and overwhelming AChEI and BChEI potentials of compounds 1–4 which reflect their dominant role in AD. Moreover, the compounds also scavenge DPPH and ABTS free radicals to a moderate level which supplement their use for AD in combination with anticholinesterase potentials. Further synthesis and biological evaluation is in progress in our laboratory.

## Methods

### Synthesis of compounds (1–4)

To a small reaction vial containing DCM (1 M) was added α−ketoester (ethyl 2-oxocyclopentanecarboxylate or ethyl 2-oxocyclohexanecarboxylate, 2 mmol). Then added organocatalyst (creatinine or 8-hydroxyquinoline, 20 mol %) and potassium hydroxide (20 mol %) and start stirring. After 2 min of stirring, added maleimide (*N*-phenyl or *N*-benzylmaleimide, 1 mmol). The progress of reaction was monitored by thin layer chromatography. At complete conversion, the reaction mixture was diluted with water (15 mL) and extracted with dichloromethane (3 × 15 mL). All the organic layers were combined and dried with anhydrous sodium sulfate (Na_2_SO_4_), filtered and concentrated at reduced pressure using rotary evaporator. The crude reaction mixture was then purified by column chromatography.

### Anticholinesterase assays

The acetylcholinesterase (AChE) from Electric eel and butyrylcholinesterase (BChE) from equine serum were used in the determination of anticholinesterase assays of compounds 1–4. The assay is based on the acetylcholinesterase promoted hydrolysis of acetylthiocholine iodide and butyrylthiocholine iodide by butyrylcholinesterase. The hydrolysis processes give 5-thio-2-nitrobenzoate anion followed by complexation with DTNB (which give yellow color) and is detected by the spectrophotometer [30].

### Preparation of solutions

Compounds (each separately) were dissolved in phosphate buffer (0.1 M) in concentrations ranging from 250–1000 μg/mL. For the preparation of 0.1 M and 8.0 ± 0.1 PH phosphate buffer solution, K_2_HPO_4_ (17.4 g/l) and KH_2_PO_4_ (13.6 g/l) were prepared and were mixed in 94 % and 6 % ratio respectively. Finally, potassium hydroxide was used to adjust pH. AChE (518 U/mg solid) and BChE (7–16 U/mg) were diluted in freshly prepared buffer pH 8.0 until final concentrations of 0.03 U/mL and 0.01 U/mL were obtained. Solutions of DTNB (0.0002273 M), ATchI and BTchI (0.0005 M) were prepared in distilled water and were kept in Eppendorf caps in the refrigerator. Galanthamine (Positive control) was dissolved in methanol and aforementioned dilutions were prepared [31].

### Spectroscopic analysis

For each assay, an enzyme solution of 5 μl was added to the cuvette followed by addition of compound (205 μl), and finally DTNB reagent (5 μl). The solution mixture was maintained at 30 °C for 15 min using water bath with subsequent addition of substrate solution (5 μl). A double beam spectrophotometer was used to measure the absorbance at 412 nm. Negative control contained all components except compounds, whereas positive control galanthamine (10 μg/mL) was used in the assay as standard cholinesterase inhibitor. The absorbances along with the reaction time were taken for four minutes at 30 °C and were repeated in triplicate. Finally, the enzyme activity and enzyme inhibition by control and tested samples were calculated from the rate of absorption with change in time (V = ΔAbs /Δt) as follow;

% enzyme inhibition = 100 - percent enzyme activity

$$ \%\kern0.5em  enzyme\kern0.5em  enzme\kern0.5em  activity=100\kern0.5em \times \kern0.5em \frac{V}{V_{\max }} $$

Where V_max_ is enzyme activity in the absence of inhibitor drug.

### DPPH free radical scavenging assay

The compounds’ free radical scavenging ability was evaluated using 1,1-diphenyl, 2-picrylhydrazyl (DPPH) free radicals [32]. Different dilutions (250, 500 and 1000 μg/mL) of compounds (0.1 mL) were added to 0.004 % methanolic solution of DPPH. After 30 min the absorbance was measured at 517 nm using UV spectrophotometer. Ascorbic acid was used as positive control.

Percent scavenging activity was calculated as;

$$ \left[\frac{A_0-{A}_1}{A_0}\right]\times \kern0.5em 100 $$

Where A_0_ is absorbance of control and A_1_ is the absorbance of the compound sample. Each experiment was performed in triplicate and inhibition curves were constructed using the GraphPad prism program (GraphPAD, San Diego, California, USA) and median inhibitory concentrations (IC_50_) values were determined.

### ABTS free radical scavenging assay

ABTS (2, 2-azinobis [3-ethylbenzthiazoline]-6-sulfonic acid) free radicals were also used to confirm the antioxidant activity of the synthesized compounds. The activity is based on the capacity of antioxidants to scavenge ABTS^+^ radical cation causing a reduction in absorbance at 734 nm. ABTS (7 mM) and K_2_S_2_O_4_ (2.45 mM) solutions were prepared and mixed. The resultant mixture was maintained at room temperature in dark for 12–16 h to get dark color solution containing ABTS^+^ radical cations. At the time of activity, ABTS^+^ radical cation solution was diluted with Phosphate buffer (0.01 M) pH 7.4 to adjust an absorbance value of 0.70 at 734 nm. Radical scavenging capability of the compounds was analyzed by mixing 300 μl of compound with 3.0 mL of ABTS solution in cuvette. The reduction in absorbance was measured spectrophotometrically after one minute of mixing the solutions and continued for six min. Ascorbic acid was used as a positive control. The assay was repeated three times and percent inhibition was calculated with formula;

$$ \%\kern0.5em \mathrm{scavenging}\kern0.5em \mathrm{effect}\kern0.5em =\kern0.5em \frac{\mathrm{Control}\kern0.5em \mathrm{absorbance}\hbox{-} \mathrm{Sample}\kern0.5em \mathrm{absorbance}}{\mathrm{Control}\kern0.5em \mathrm{absorbance}}\kern0.5em \times 100 $$

The antioxidant effect was expressed in terms of percent inhibition and as EC_50_ (compounds concentration required for 50 % reduction of ABTS radicals).

### Estimation of IC_50_ values

Concentrations of the compounds that inhibited substrate hydrolysis (AChE and BChE) by 50 % (IC_50_). Radical scavenging ability was calculated by a linear regression analysis among the inhibition percentages against the compounds concentrations via the Excel program.

### Statistical analysis

Data is represented as mean ± SEM, *n* = 3. Two-way ANOVA followed by Bonferroni test was applied for significant difference between standard drugs and test samples at 95 % confidence interval. Values were considered significantly different with * *P* < 0.05, ** *P* < 0.01 and *P* < 0.001. ns: Test compounds non significantly different in comparison to standard drug *P* > 0.05.

## Additional file

Additional file 1:
**The** ^**1**^ **H &** ^**13**^ **C NMRs of the synthesized compounds are provided.**

## Abbreviations

AD: Alzheimer’s disease
AChE: Acetylcholinesterase
BChE: Butyrylcholinesterase
AChEI: Acetylcholinesterase inhibition, BChEI, butyrylcholinesterase inhibition
DPPH: 1,1-diphenyl 2-picrylhydrazyl
ABTS: 2,2-azinobis[3-ethylbenzthiazoine]-6-sulfonic acid
ROS: Reactive oxygen species, IC_50_, median inhibitory concentration
SEM: Standard error mean
