# Supplementary material for: Synthesis, anticholinesterase and antioxidant potentials of ketoesters derivatives of succinimides: a possible role in the management of Alzheimer’s
Source: Chem Cent J. 2015 May 26;9:31. doi: 10.1186/s13065-015-0107-2 (PMC4461796; doi:10.1186/s13065-015-0107-2)

**Additional data:**

**Chemicals and Drugs:**

The chemicals and drugs used in this research work are given below;

**α-ketoesters:**

- Ethyl-2-oxocyclopentane carboxylate (Sigma Aldrich Cat. No. 168092)
- Ethyl-2-oxocyclohexane carboxylate (Sigma Aldrich Cat. No. E20004)

**Maleimides:**

- *N*-Phenylmaleimide (Sigma Aldrich Cat. No. P27100)
- *N*-Benzylmaleimide (Sigma Aldrich Cat. No. 408018)

**Catalyst components:**

- Creatinine
- 8-Hydroxyquinoline
- Potassium hydroxide (Sigma Aldrich Cat. No. P1767)

**Solvents:**

- Dichloromethane
- n-Hexane
- Ethyl acetate

**Enzymes and antioxidant materials:**

- AChE Electric eel (type-VI-S, CAS 9000-81-1 Sigma-Aldrich GmbH USA)
- BChE equine serum Lyophilized (CAS 9001-08-5 Sigma-Aldrich GmbH USA)
- Acetylthiocholine Iodide (CAS1866-15-5 Sigma-Aldrich UK)
- Butyrylthiocholine Iodide CAS 2494-56-6 Sigma-Aldrich Switzerland)
- 5,5-dithio-bis-nitrobenzoic acid (DTNB) (CAS 69-78-3 Sigma-Aldrich Germany)
- Galanthamine hydrobromide Lycoris Sp. (CAS 1953-04-4 Sigma-Aldrich France)
- Di-potassium hydrogen phosphate (K_2_HPO_4_)
- Potassium di-hydrogen phosphate (KH_2_PO_4_)
- DPPH (CAS 1898-66-4 Sigma Aldrich)
- ABTS (CAS 30931-67-0 Sigma Aldrich)
- K_2_S_2_O_4_ (Riedel-de Haen Germany)
- Gallic acid (CAS 149-91-7 GmbH USA)
- Folin Ciocalteu reagent (FCR) Merck Co. (Germany)

**^1^H NMR spectrum of compound 1**
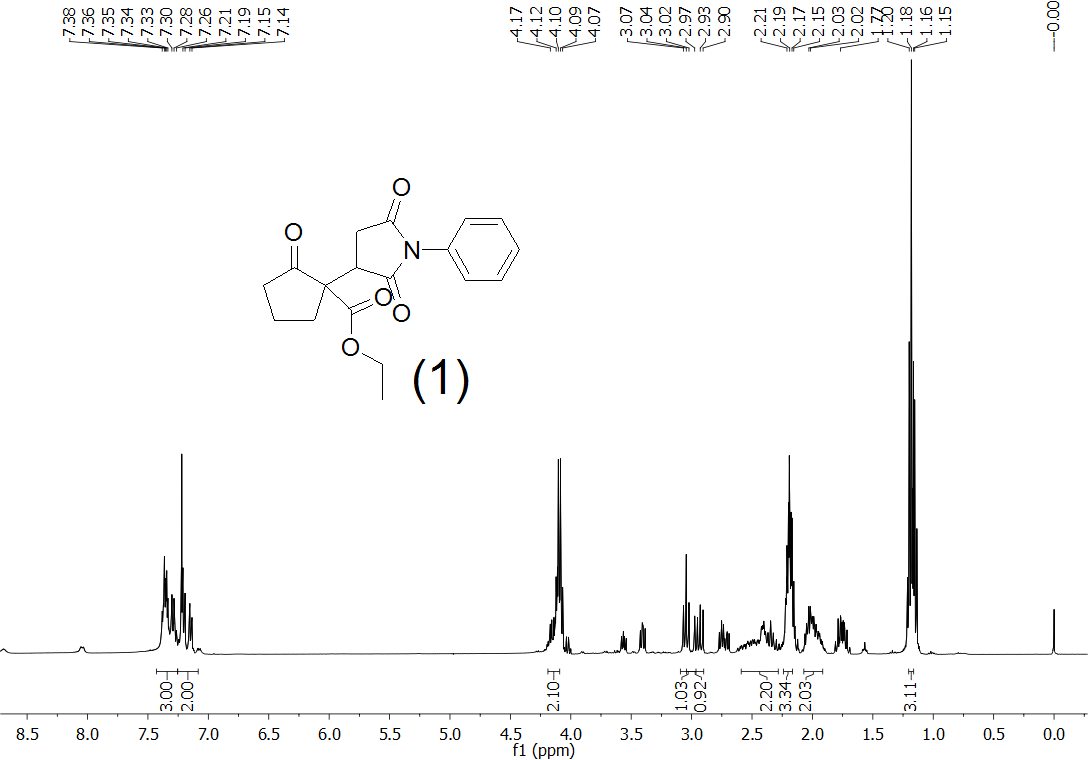


**^13^C NMR of compound 1**


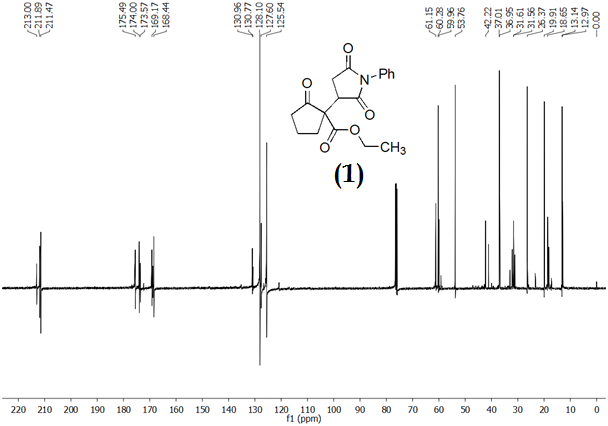


**^1^ H NMR spectrum of compound 2**


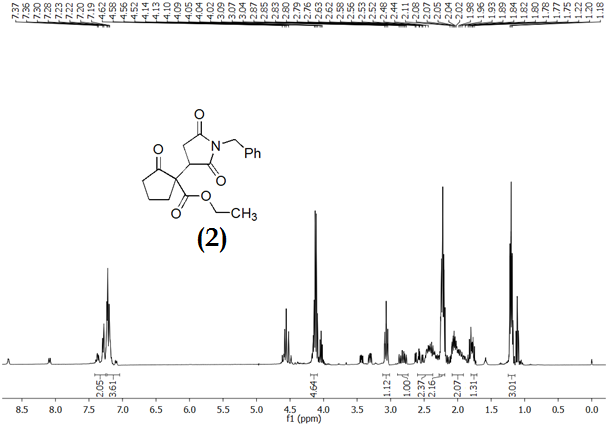


**^13^C NMR spectrum of compound 2**


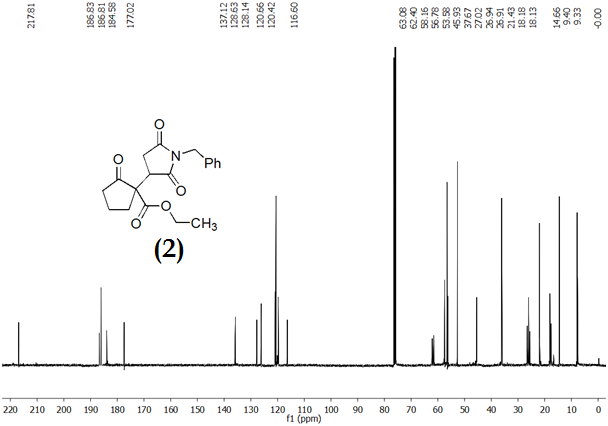


**^1^H NMR spectrum of compound 3**

**
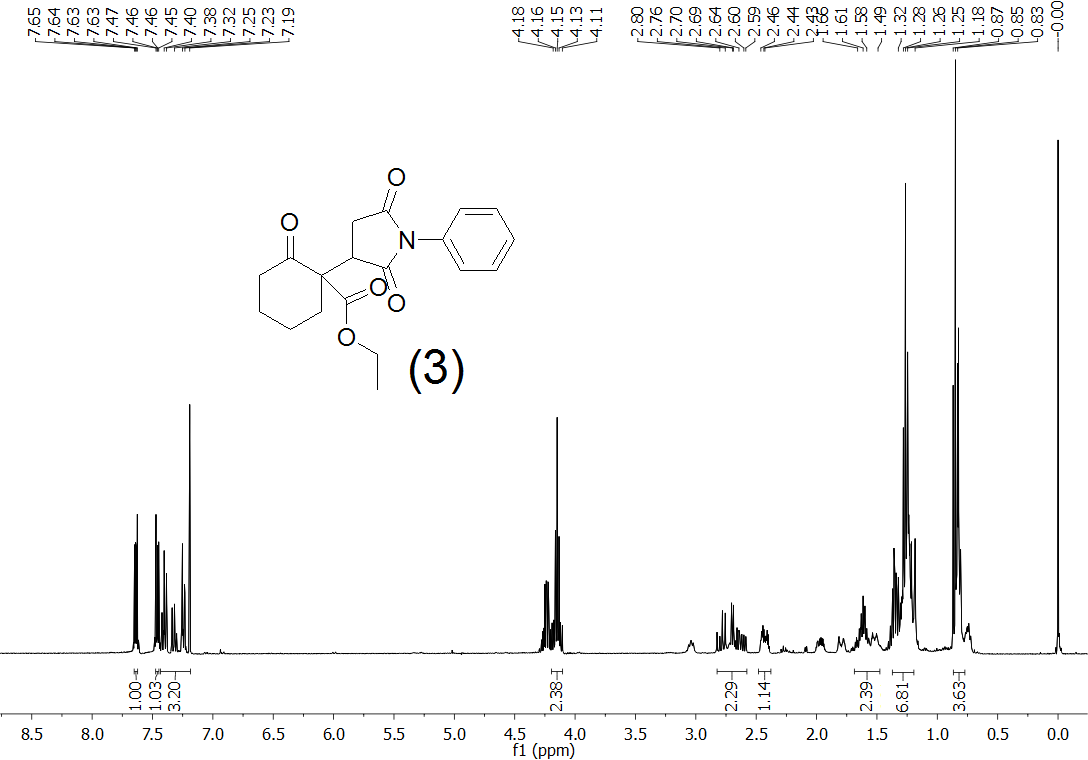
**

**^13^C NMR spectrum of compound 3**


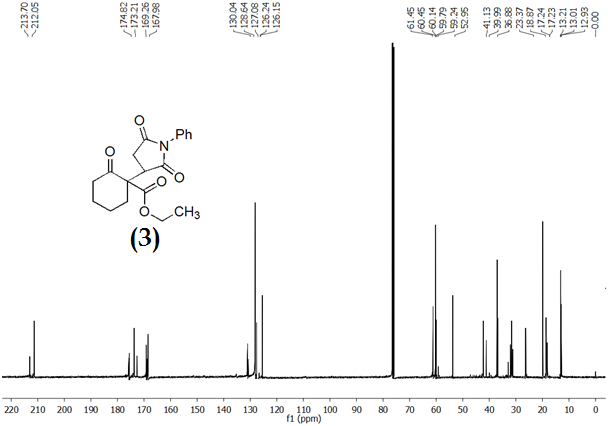


**^1^H NMR spectrum of compound 4**


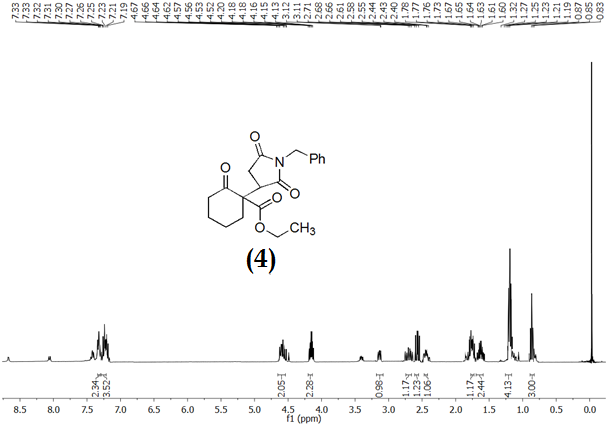


**^13^C NMR spectrum of compound 4**


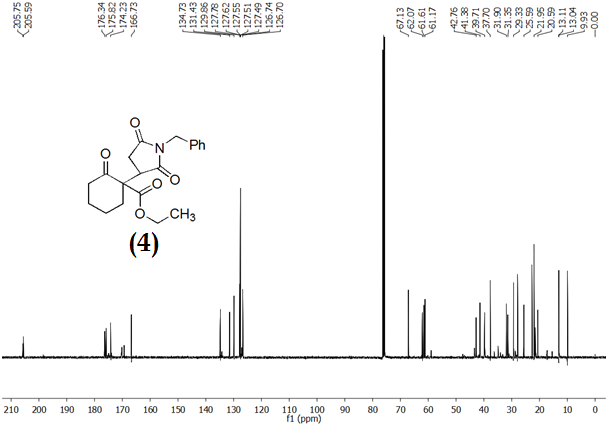

Supplement: Additional file 1: — The 1 H & 13 C NMRs of the synthesized compounds are provided. [file 13065_2015_107_MOESM1_ESM.doc]
